# Maternal near miss and potentially life-threatening condition determinants in patients with type 1 diabetes mellitus at a university hospital in São Paulo, Brazil: a retrospective study

**DOI:** 10.1186/s12884-020-03392-y

**Published:** 2020-11-10

**Authors:** Luiza Russo de Morais, Beatriz Costa Patz, Felipe Favorette Campanharo, Patricia Médici Dualib, Sue Yazaki Sun, Rosiane Mattar

**Affiliations:** 1 Obstetrics Department, Universidade Federal de São Paulo (UNIFESP) Escola Paulista de Medicina, São Paulo, 04021-001 Brazil; 2 Endocrinology Department, Universidade Federal de São Paulo (UNIFESP) Escola Paulista de Medicina, São Paulo, 04021-001 Brazil

**Keywords:** Maternal near miss, Type 1 diabetes mellitus, Severe maternal morbidity, Potentially life-threatening condition, Maternal health

## Abstract

**Background:**

To date, the rates of potentially life-threatening condition (PTLC), maternal near miss (MNM) and maternal deaths in pregnant patients with type 1 diabetes mellitus (T1DM) and variables associated to it have not been studied.

**Methods:**

This study was as a cross-sectional retrospective study conducted at São Paulo Hospital of Universidade Federal de São Paulo, a tertiary hospital that provides public medical care through the Brazilian unified health system to high-risk pregnancies. Inclusion criteria were T1DM pregnant women who delivered from January 2005 to December 2015. Three groups were established by the World Heath Organization criteria and associations were assessed using the chi-square test in between MNM and no morbidity or PLTC and no morbidity. A *P* value < 0.05 was considered statistically significant.

**Results:**

The final sample included 137 patients, 8 MNM cases (5.84%), 51 PLTC (37.23%), no cases of maternal deaths and 78 patients (56.93%) did not present any complication. Moreover, there were 122 live births, resulting in a near miss rate of 65.5 per 1.000 live births in patients with T1DM. Two of the MNM cases were for clinical criteria (uncontrollable fit in both) and laboratory criteria for the other six: one patient with severe acute azotemia (creatinine > 300 μmol/ml), one patient with severe hypoperfusion (lactate > 5 mmol/L) and four of them with loss of consciousness and the presence of glucose and ketoacids in urine. PLTC criteria were studied in MNM and PLTC cases. Prolonged hospital stay was the most prevalent PLTC criteria in both groups (100% of MNM cases and 96% of PLTC), followed by renal failure in 50% of MNM cases and severe preeclampsia in 22% of PLTC cases. This study could not find any association between prenatal factors or sociodemographic characteristics with maternal morbidity.

**Conclusions:**

MNM rate in T1DM was extremely high, and determined by complications of the primary disease or hypertensive disorders. No sociodemographic variables studied were related to maternal morbidity; therefore, we could not predict what increases MNM and PLTC in this specific population.

## Background

Maternal health has been widely studied in the last decades. As the World Health Organization (WHO) makes continuous efforts to reduce maternal mortality, in 2009, the Organization defined maternal near miss (MNM) as “a woman who nearly died but survived a complication that occurred during pregnancy, childbirth or within 42 days of termination of pregnancy” [1]. In the same document, potentially life-threatening conditions (PLTC) were defined as an extensive category of clinical conditions that can threaten a woman’s life during the same period and lead to MNM, and the term severe maternal morbidity (SMM) included both outcomes. This new terminology improved studies in maternal health, whereas now we can progressively study patients from PLTC to MNM and finally maternal death, identifying risk factors and implementing prophylactic measures in each step to avoid mortality.

Maternal and neonatal morbidity in patients with type 1 diabetes mellitus (T1DM) is well documented, with higher chances of preeclampsia, preterm birth, perinatal mortality, hypoglycemia, retinopathy, hypertension, nephropathy or diabetic ketoacidosis [2, 3]. On one hand these patients have reduced rate of pregnancy - partially due to lower fertility [4], but on the other hand the prevalence of T1DM is increasing among the youth [5]. Therefore, the tendency is to increase the number of pregnant with T1DM and obstetricians have to be aware of severe outcomes and complications of these patients. It is important to highlight that, particularly in Brazil, the elevated economic costs [6] and patients educational conditions needed to treat T1DM can compromise adequate use of insulin and medical supplies, resulting in higher chances of chronic complications. In these cases, patients tend to get pregnant without controlling the baseline disease, so that obstetric care becomes even more challenging.

To date, several studies have been published about MNM in Brazil and worldwide, but none of them has studied exclusively T1DM. We believe that SMM in T1DM can result from complications of the pregnancy itself, diabetes or other causes – such as prenatal care or sociodemographic characteristics. Therefore, the aim of this study was to investigate what variables could determine SMM in T1DM and the rate of MNM, PLTC and maternal mortality.

## Methods

### Design, setting and aim of the study

This was a cross-sectional retrospective study conducted at São Paulo Hospital of Universidade Federal de São Paulo (UNIFESP), São Paulo, Brazil. This is a tertiary university hospital that provides universal care to high-risk pregnant patients through the Brazilian unified health system, free of charge. The aim of the study was to determine rates of PTLC, MNM and maternal deaths in T1DM and to identify which variables were related to these outcomes.

### Study population and selection

Patients included were T1DM pregnant women who had their deliveries at Hospital São Paulo from January 2005 to December 2015 regardless if they had received prenatal care at UNIFESP or elsewhere. Exclusion criteria were cases with missing data. We considered a ten-year study period convenient to retrieve a significant sample. The outcome studied was PLTC, MNM, maternal death or no morbidity. PLTC and MNM cases were identified according to WHO criteria [7], using data collected from patient medical records.

### Data collection

Retrospective data was collected in 2017 from medical records of all patients. Data collection was conducted separately by two researchers and revised by both.

### Definition of variables

Variables studied were socioeconomic (age, schooling level, self-reported skin color, conjugal situation) obstetric history (parity and number of previous deliveries) pregnancy-related variables (number of prenatal appointments and initial glycated hemoglobin) and obstetric outcome (miscarriage or type of delivery and gestational age).

### Statistical analysis

Patients were assessed in 3 groups: MNM, PLTC and no morbidity. The first two groups were determined by descriptive analysis of the WHO criteria [7] and patients who did not fill any criterion for those groups were considered without morbidity. Patients that filled mutually criteria for MNM and PLTC were considered exclusively as MNM cases. All variables previously elucidated were researched in the groups and associations were assessed using the chi-square test in between: MNM and no morbidity or PLTC and no morbidity. A *P* value < 0.05 was considered statistically significant. Statistical data were analyzed using Microsoft Office Excel version 2010, Microsoft, Washington, United States.

## Results

In a ten-year period, from 2005 to 2015, there were 142 T1DM pregnant women who delivered at Hospital São Paulo*.* Five patients had missing data and were excluded, resulting in a final sample of 137 patients. Among these, 8 were MNM cases (5.84%), 51 were PLTC cases (37.23%) and 78 did not present any complications and were included in the “no morbidity “group (56.93%). There were no cases of maternal deaths. Moreover, there were 122 live births, resulting in a near miss rate of 65.5 per 1000 live births in patients with T1DM.

Regarding sociodemographic characteristics: self-reported skin color, age, scholarly or civil status did not present statistical significance in between the groups (*P* > 0.05) (Table 1). However, in all groups, most of our patients were white and had studied from 11 to 14 years. Even though it was not statistically relevant, most of the patients with MNM had ages in between 25 and 35 (62.5%), whereas in the other two groups patients were younger than 25. Prenatal care and obstetric history were also studied, but the only significant difference was found in between nuliparity and PLTC (*P* = 0.03). There was no association of MNM or PLTC with the number of prenatal appointments (*P* = 0.63 and *P* = 0.91, respectively) neither with the initial value of glycated hemoglobin (*P* = 0.36 for both groups).

**Table 1:** Sociodemografic and obstetric characteristics associated with maternal morbidity in patients with type 1 diabetes

Characteristic	MNM (%)	PLTC (%)	No morbidity (%)	***P*** value*	
				MNM**	PLTC***
**Age**					
15–25 years	37.5	62.76	56.41	0.31	0.71
26–35 years	62.5	31.37	38.46		
≥ 35 years	0	5.88	5.13		
**Self-reported skin color**					
White	62.5	66,6	58.9	1.00	0.8
Not White	37.5	33.3	41.1		
**Schooling level**					
≤ 7 years	12.5	18.42	20.5	0.54	0.38
8–10 years	12.5	40	29.5		
11–14 years	75	39.22	40		
≥ 15 years	0	0	9		
**With partner**					
Yes	50	47.06	51.28	0.9	0.63
No	50	52.94	48.72		
**Primiparous**					
Yes	50	49,2	46,15	0,77	0,44
No	50	45,1	53,85		
**Number of previous deliveries**					
None	87.5	82.35	65.38	0.21	0.03
1 or more	12.5	17.65	34.62		
**Number of prenatal visits**					
None	12.5	5.88	6.41	0.63	0.91
1–6	50	23.53	21.79		
6 to 12	37.5	47.06	42.31		
13 to 18	0	19.61	21.79		
> 18	0	3.92	7.69		
**Initial glycated hemoglobin**					
Missing data	0	9.8	10.25	0.36	0.36
< 7,2	25	19.61	14.1		
7,2 – 9,1	25	27.45	26.92		
9,2 – 11,1	37.5	11.76	12.82		
> 11,1	12.5	31.37	35.9		

*MNM* maternal near miss, *PLTC* potentially life-threatening conditions, * chi-square test, ** *P* value comparing variable of maternal near miss cases with patients with no morbidity, *** *P* value comparing variable of potentially life-threatening conditions cases with patients with no morbidity

Pregnancies with MNM or PLTC had significant earlier gestational age resolution in comparison with patients without complications (*P* < 0.001 and *P* = 0.03, respectively) (Table 2). However, type of delivery was only relevant on patients with PLTC, with higher rates of C-sections (*P* = 0.048).

**Table 2:** Obstetric outcome associated with severe maternal morbidity or not in patients with type 1 diabetes

Characteristic	MNM (%)	PLTC (%)	No morbidity (%)	***P*** value *	
				MNM**	PLTC***
**Gestational age at resolution**					
< 20 weeks	12.5	7.84	7.69	< 0.001	0.03
20–27 weeks	12.5	7.84	5.13		
28–33 weeks	62.5	17.65	7.69		
34–37 weeks	0	43.14	28.21		
> 37 weeks	12.5	23.53	28.21		
**Pregnancy outcome**					
Miscarriage	12.5	1.96	12.5	0.5	0.048
C-section	50	72.55	75		
Vaginal	37.5	19.61	12.5		

*MNM* maternal near miss, *PLTC* potentially life-threatening conditions, * chi-square test, ** *P* value comparing variable of maternal near miss cases with patients with no morbidity, *** *P* value comparing variable of potentially life-threatening conditions cases with patients with no morbidity, underlined *P* value statistically significant

There were 8 cases of maternal near miss. Two of them for clinical criteria (uncontrollable fit in both), other two for management criteria (dialysis due acute renal failure in both) and laboratory criteria for the six others. Laboratorial criteria were: one patient with severe acute azotemia (creatinine > 300 μmol/ml or > 3.5 mg/dL), one patient with severe hypoperfusion (lactate > 5 mmol/L or > 45 mg/dL) and four with loss of consciousness and the presence of glucose and ketoacids in urine.

**Fig. 1:**
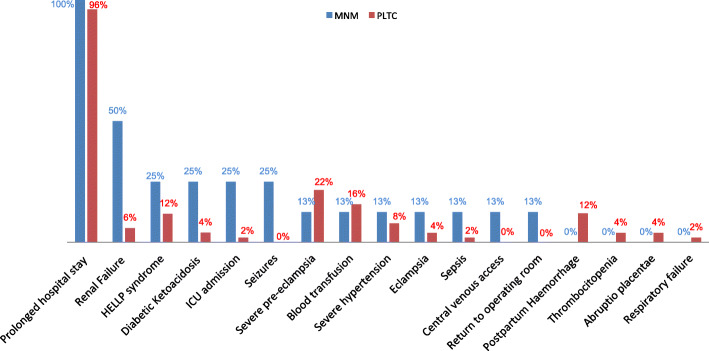
Percentage of PTLC criteria in PLTC and MNM cases in pregnant with type 1 diabetes. MNM: maternal near miss, PLTC: potentially life-threatening conditions, HELLP: syndrome hemolysis, elevated liver enzymes, low platelet count; ICU: intensive care unit

It is known that MNM progress from a PLTC case. Therefore, we studied into the cases of MNM what criteria of PLTC were previously onsets. Figure 1 exhibits percentages of PLTC criteria in PLTC cases versus MNM cases. Prolonged hospital stay was the most prevalent PLTC criterion in our population (present in 100% of MNM and in 96% of PLTC cases), followed by renal failure in half of patients with MNM and severe preeclampsia in 22% of patients with PLTC.

## Discussion

MNM ratio was 65.5 per 1000 live births in patients with T1DM. This rate is largely variable on literature depending on the geographic region, hospital and obstetric care, but it is known that, in comparison to other studies and populations, this is an extremely high number. A national Brazilian study [8] reported a MNM ratio of 10.2, while a study with high-risk pregnancies reported a rate of 54.8 [9]. To the best of our knowledge, SMM in T1DM has not yet been studied. We presume that this rate is so high in our population because of the burden of this autoimmune disease. However, there were no cases of maternal deaths, evidencing that, even though there is a high morbidity, patients that receive proper care in a tertiary center do not evolve to mortality.

Half of our patients with MNM had renal insufficiency. This is a common complication of T1DM during pregnancy that underlines the burden of the disease, since diabetic nephropathy is associated with a 2–4 fold increased risk of preeclampsia, preterm delivery and perinatal death [10]. A multidisciplinary team should monitor patients with previous diabetic nephropathy, before and during pregnancy, to reduce morbidity. Another important complication in MNM was diabetic ketoacidosis, as it occurred in 2,9% of our patients (4/137). The overall incidence of ketoacidosis in T1DM is uncertain and lacks recent data, with the overall incidence in all diabetic pregnancies varying from 1 to 10% [11, 12]. In order to reduce these complications, we suggest frequent prenatal appointments focusing on glycemic control and correct insulin use.

In our population there were a significant percentage of patients with SMM that presented some kind of hypertensive disorder. Similarly, in the Brazilian general population, severe preeclampsia is the most important cause of near miss [13, 14], and this is even more relevant in our group because T1DM increases the odds of preeclampsia. On this research use of aspirin was not studied, but we assume that it was prescribed to all patients who received prenatal care at our institution, since it is part of prenatal protocol in patients with T1DM. It is a consent in the medical community that low-dose aspirin should be prescribed from the end of the first trimester until the baby is born in order to lower the risk of preeclampsia in patients with T1DM [15]. Our findings reinforce this recommendation due to the morbidity and prevalence of preeclampsia in this population.

Other important PLTC criteria were hemorrhagic complications and blood transfusions, complications than increase maternal morbidity worldwide [16]. These findings could be explained by the high rate of C-section, 72.5% among patients with PLTC, since the procedure increases the risk of blood loss and postpartum hemorrhages. In contrast, the C-sections rate on MNM patients was 56.42%, similar to the national statistic of 57% [17] and these patients had less hemorrhagic complications, reinforcing our theory. The only case of blood transfusion in MNM was in the miscarriage. We consider that this complication can be minimized if C-section rate is reduced.

Almost all of the patients with PLTC were hospitalized for more than 7 days. We believe that this condition is due not only to the disease and obstetric complications previously discussed, but also to achieve an appropriate glycemic control, that can be challenging during the pregnancy and puerperium. In the last couple of years, the use of technology has improved communication in between the patient and the endocrinology team to control the insulin bomb, which is currently done remotely. However, in our population, this has been mostly used during the last 5 years and a large part of our cases date previously. Currently, this barrier has been unraveled and prolonged hospital stay would likely occur in a smaller percentage of our cases. It should be noted that prolonged hospitalization is a PLTC criterion, but discharging patients only after adequate glycemic control may minimize infectious postoperative complications and readmissions to the hospital. There is recent data that indicates that specifically T1DM retains increased risk for readmission, with even higher risks for those with public insurance [18].

This study could not find any relation between prenatal factors - such as number of visits and obstetric history - or sociodemographic characteristics with maternal morbidity. Therefore, we could not predict any factor that increases morbidity in this specific population. Less than six prenatal visits have been related to increased risk of maternal near miss in Brazil [8], but we could not find this relation. Six prenatal visits are recommended to low risk pregnancies and probably a higher cutoff in high-risk cases like T1DM would be found with further analysis. Despite not having statistical significance in this study, we noticed that in the group without morbidity 9% of the patients had studied more than 15 year, but this did not occur on the other two groups. Maternal morbidity appears to be inversely proportional to years of study in T1DM.

T1DM is a public health challenge in Brazil. Insulin administration procedure requires that the patient understands the whole process, relates glycemic levels to insulin units and applies it correctly. This can be challenging depending on patient’s educational level, that it is not high in our population – as evidenced in our results. Brazilian government measures to reduce maternal morbidity in this group include free insulin supplies and tertiary centers with multidisciplinary teams with prenatal assistance. These measures tend to decrease complications of the disease but can be insufficient since controlling T1DM depends on multiple factors.

Another aspect studied was glycated hemoglobin at the first trimester but no relation was found between high glycated hemoglobin at first trimester and maternal morbidity in this study. We infer that patients with higher levels of glycated hemoglobin were the ones with miscarriages, since this relation was previously demonstrated [19], and this outcome occurred similarly in between the groups. The value of glycated hemoglobin translates as a picture of the patient’s control of the baseline disease at that trimester. Patients that presented high levels of glycated hemoglobin in the first trimester probably acknowledged at that time the importance of glycemic control to the unborn’s health. So, it could be that our patients had their best medical care and glycemic control during pregnancy since the concern with the fetus may motivate her self care. Additionally it is important to highlight the importance of referring T1DM pregnant women to tertiary hospitals with specialized multidisciplinary team to improve their care during pregnancy.

Finally, regarding gestational age of delivery, we observed that MNM and PLTC determined more premature births. There is a positive correlation in between maternal near miss and neonatal near miss in patients with T1DM [20]. This is probably due to maternal complications such as preeclampsia and renal failure previously discussed, whereas maternal morbidity increases premature birth and, therefore, neonatal morbidity. If maternal health is diminished by actions previously discussed neonatal complications will decline.

The main limitation of the study is mostly its retrospective design. Since data was collected from medical records, some criteria were difficult to identify like cyanosis or initial creatinine, and it would be interesting to relate this last variable to maternal morbidity since a large number of our cases evolved to renal complication. Moreover, we suggest a prospective study with a larger sample to clarify our associations.

The strength of the study is that it pioneers in analyzing the relation between severe maternal mortality and T1DM in Brazil and that its conclusions can improve medical assistance in patients with T1DM.

MNM was extremely high in patients with T1DM. This group should have preconception counseling to be advised about gestational risks and encouraged to plan pregnancy once her baseline disease is well managed. Clinicians should be aware that these patients are at higher chances of hypertensive disorders, renal failure, and ketoacidosis, and that, for now, we could not identify variables associated with maternal morbidity. Therefore, we consider that all pregnant patients with T1DM should be considered as a potential case of SMM and cared mutually by obstetricians and endocrinologists. During delivery, we recommend monitoring blood pressure, efforts to avoid prematurity and postpartum hemorrhages to decrease PLTC cases. Finally, it is important to highlight that these patients do have higher hospitalization periods, and that, paradoxically, this might lead to less morbidity.

## Conclusion

MNM rate in T1DM was extremely high and determined by complications of the primary disease or hypertensive disorders. Currently, we could not predict those who will present higher morbidity, so all patients with T1DM should be carefully assisted, as SMM is expressive in this population.

## Data Availability

Excel data used to support the findings of this study are available from the corresponding author upon request.

## Abbreviations

WHO: World Health Organization
MNM: Maternal near miss
PLTC: Potentially life-threatening conditions
SMM: Severe maternal morbidity
T1DM: Type 1 diabetes mellitus
UNIFESP: Universidade Federal de São Paulo

## References

[1] World Health Organization (2009). Report on the World Health Organization working group on the classification of maternal deaths and severe maternal morbidities. Bull World Health Organ.

[2] McCance DR, Casey C. Type 1 diabetes in pregnancy. Endocrinol Metab Clin North Am. 2019. 10.1016/j.ecl.2019.05.008. 10.1016/j.ecl.2019.05.008 31345519

[3] Ringholm L, Mathiesen ER, Kelstrup L, Damm P. Managing type 1 diabetes mellitus in pregnancy – from planning to breastfeeding. Nat Rev Endocrinol. 2012. 10.1038/nrendo.2012.154. 10.1038/nrendo.2012.154 22965164

[4] Lin YH, Chen KJ, Peng YS, Chen PC, Yang YH. Type 1 diabetes impairs female fertility even before it is diagnosed. Diabetes Res and Clin Practi. 2018. 10.1016/j.dia.bres.2018.07.010. 10.1016/j.diabres.2018.07.010 30003941

[5] Mayer-Davis EJ, Dabelea D, Lawrence JM. Incidence trends of type 1 and type 2 diabetes among youths, 2002-2012. N Engl J Med. 2017. 10.1056/NEJMc1706291. 10.1056/NEJMc1706291 PMC5639715 28723318

[6] Cobas RA, Ferraz MB, Matheus ASMM, Tannus LRM, Negrato CA, Araujo LA, et al. The cost of type 1 diabetes: a nationwide multicentre study in Brazil. Bulletin of the World Health Organ. 2013. 10.2471/BLT.12.110387. 10.2471/BLT.12.110387 PMC3777141 24052680

[7] Say L, Souza JP, Pattinson RC (2009). Maternal near miss—towards a standard tool for monitoring quality of maternal health care. Best Pract Res Clin Obstet Gynaecol.

[8] Domingues RMSM, Dias MAB, Schilithz AOC, Leal MDC (2016). Factors associated with maternal near miss in childbirth and the postpartum period: findings from the birth in Brazil National Survey, 2011–2012. Reprod Health.

[9] De Lima THB, Amorim MM, Kassar SB, Katz L. Maternal near miss determinants at a maternity hospital for high-risk pregnancy in northeastern Brazil: a prospective study. BMC Pregnancy Childbirth. 2019. 10.1186/s12884-019-2381-9. 10.1186/s12884-019-2381-9 PMC6670122 31370813

[10] Mathiesen ER, Ringholm L, Feldt-Rasmussen B, Clausen P, Damm P. Obstetric nephrology: pregnancy in women with diabetic nephropathy – the role of antihypertensive treatment. Clin J Am Soc Nephrol. 2012. 10.2215/CJN.00920112. 10.2215/CJN.00920112 22917698

[11] Ramin KD (1999). Diabetic ketoacidosis in pregnancy. Obstet Gynecol Clin N Am.

[12] Parker JA, Conway DL. Diabetic ketoacidosis in pregnancy. Obstet Gynecol Clin North Am. 2007. 10.1016/j.ogc.2007.08.001. 10.1016/j.ogc.2007.08.001 17921013

[13] Cecatti JG, Souza JP, Neto AFO, Parpinelli MA, Souza MH, Say L, Pattison RC. Pre-validation of the WHO organ dysfunction based criteria for identification of maternal near miss. Reprod Health. 2011. 10.1186/1742-4755-8-22. 10.1186/1742-4755-8-22 PMC3162482 21810265

[14] Zanette E, Parpinelli MA, Surita FG, Costa ML, Haddad SM, Sousa ML et.al. Brazilian network for surveillance of severe maternal morbidity group maternal near miss and death among women with severe hypertensive disorders: a Brazilian multicenter surveillance study. Reprod Health 2014;11(1):4. 10.1186/1742-4755-11-4 PMC3896751 24428879

[15] American Diabetes Association. Management of Diabetes in Pregnancy: Standards of Medical Care in Diabetes. Diabetes Care. 2019. 10.2337/dc19-S014.

[16] Say L, Chou D, Gemmill A, Tunçalp O, Moller AB, Daniels J, et al. Global causes of maternal death: a WHO systematic analysis. Lancet Glob Health. 2014. 10.1016/S2214-109X(14)70227-X. 10.1016/S2214-109X(14)70227-X 25103301

[17] Ministério da Saúde, Saúde Brasil 2014 – Uma análise da situação de saúde e de causas externas. 2015. http://bvsms.saude.gov.br/bvs/publicacoes/saude_brasil_2014_analise_situacao.pdf. Accesed in 16 June 2020.

[18] Mourad M, Wen T, Friedman AM, Lonier JY, DʼAlton ME, Zork N. Postpartum readmissions among women with diabetes. Obstet Gynecol. 2019. 10.1097/aog.0000000000003551. 10.1097/AOG.0000000000003551 PMC6923572 31809421

[19] Kekäläinen P, Juuti M, Walle T, Laatikainen T (2016). Pregnancy planning in type 1 diabetic women improves glycemic control and pregnancy outcomes. J Matern Fetal Neonatal Med.

[20] Morais LR, Patz BC, Campanharo FF, Dualib PM, Sun SY, Mattar R. Neonatal near miss among newborns of women with type 1 Diabetes Mellitus. Obstet Gynecol Int. 2019. 10.1155/2019/8594158. 10.1155/2019/8594158 PMC6701311 31467554

